# A Common Missense Variant Causing Factor XI Deficiency and Increased Bleeding Tendency in Maine Coon Cats

**DOI:** 10.3390/genes13050792

**Published:** 2022-04-28

**Authors:** Henrike Kuder, S. Kent Dickeson, Marjory B. Brooks, Alexandra Kehl, Elisabeth Müller, David Gailani, Urs Giger

**Affiliations:** 1 Vetsuisse Faculty, University of Zürich, Winterthurerstrasse 260, CH-8057 Zurich, Switzerland; henrike.kuder@uzh.ch; 2 Laboklin GmbH & Co. KG (Labogen), Steubenstrasse 4, D-97688 Bad Kissingen, Germany; kehl@laboklin.com (A.K.); mueller@laboklin.com (E.M.); 3 Department of Pathology, Microbiology and Immunology, Vanderbilt University Medical Center, 1301 Medical Center Dr, Nashville, TN 37232, USA; stephen.k.dickeson@vumc.org (S.K.D.); dave.gailani@vanderbilt.edu (D.G.); 4 Comparative Coagulation Laboratory, Cornell University, 240 Farrier Road, Ithaca, NY 14853, USA; mbb9@cornell.edu

**Keywords:** hemorrhage, coagulopathy, mutation, feline, protease, partial thromboplastin time

## Abstract

Hereditary factor XI (FXI) deficiency is characterized as an autosomal mild to moderate coagulopathy in humans and domestic animals. Coagulation testing revealed FXI deficiency in a core family of Maine Coon cats (MCCs) in the United States. Factor XI-deficient MCCs were homozygous for a guanine to adenine transition resulting in a methionine substitution for the highly conserved valine-516 in the FXI catalytic domain. Immunoblots detected FXI of normal size and quantity in plasmas of MCCs homozygous for V516M. Some FXI-deficient MCCs experienced excessive post-operative/traumatic bleeding. Screening of 263 MCCs in Europe revealed a mutant allele frequency of 0.232 (23.2%). However, V516M was not found among 100 cats of other breeds. Recombinant feline FXI-M516 (fFXI-M516) expressed ~4% of the activity of wild-type fFXI-V516 in plasma clotting assays. Furthermore, fFXIa-M516 cleaved the chromogenic substrate S-2366 with ~4.3-fold lower catalytic efficacy (k*_cat_*/K*_m_*) than fFXIa-V516, supporting a conformational alteration of the protease active site. The rate of FIX activation by fFXIa-M516 was reduced >3-fold compared with fFXIa-V516. The common missense variant FXI-V516M causes a cross-reactive material positive FXI deficiency in MCCs that is associated with mild-moderate bleeding tendencies. Given the prevalence of the variant in MCCs, genotyping is recommended prior to invasive procedures or breeding.

## 1. Introduction

The coagulation protein factor XI (FXI) is a serine protease zymogen found in the blood of mammals. It is synthesized in hepatocytes in placental mammals and circulates as a complex with high molecular weight kininogen [1,2,3]. Factor XI is a dimer comprised of identical 80 kDa polypeptides connected by a disulfide bond. Each subunit contains four 90 to 91 amino acid repeated domains, referred to as “apple domains” and a trypsin-like catalytic domain [2]. In primitive mammals (monotremes) and marsupials FXI is believed to be converted to the active protease FXIa by the enzyme factor XIIa (FXIIa), as part of the intrinsic pathway of coagulation. However, thrombin may be the more physiologically relevant FXI activator in placental mammals. FXIa propagates thrombin formation by converting factor IX (FIX) to FIXaβ [1].

Inherited FXI deficiency is an autosomal trait first reported in humans by Rosenthal and colleagues in 1953. The condition causes a mild to moderate bleeding disorder characterized by post-traumatic and surgical hemorrhage, particularly when involving tissues with high intrinsic fibrinolytic activity, such as the mouth, nose, and urinary tract [4,5,6,7,8,9,10]. The prevalence of severe FXI deficiency (plasma activity <15% of normal) may vary among human populations, with an estimate of 12.9 per 100,000 in Europeans [11]. The condition is particularly common in certain ethnic groups (e.g., Ashkenazi and Iraqi Jews and French Basques) [10,12,13], and has been reported more frequently in some geographic regions (e.g., Israel, France, Southern Italy, and the United Kingdom) [14,15,16,17].

Hereditary FXI deficiency has been identified in a few domesticated species, with high allele frequencies of causative mutations reported in certain breeds of cattle and dogs [18,19,20,21,22,23,24]. In these animals, as in humans, the condition is associated with a mild to moderate propensity for excessive bleeding. Here, we describe a novel common variant in the FXI gene (*F11*) in Maine Coon cats (MCC), a common domestic cat breed. The mutation results in a circulating FXI variant with altered catalytic activity and typically manifests as a trauma- or surgery-induced bleeding disorder.

## 2. Materials and Methods

### 2.1. Animals and Samples

A FXI-deficient MCC with abnormal bleeding was identified in a cattery in the United States in 2019. Subsequent coagulation testing of MCCs from this and a second cattery related to the index case revealed additional FXI-deficient animals. Individuals from these two catteries were considered the core family for this study. Primary care veterinarians for MCCs in the core family provided blood samples for coagulation testing and information regarding age, sex, and bleeding history. Client consent was obtained for sample acquisition with approval from the Institutional Animal Care and Use Committee at Cornell University. Citrated plasma samples were tested at the Comparative Coagulation Laboratory (Cornell, College of Veterinary Medicine, Ithaca, NY, USA). Cell pellets from blood samples and cheek swabs for core family members, and archived DNA from unrelated FXI-deficient MCCs were sent to Labogen (Laboklin GmbH & Co. KG, Bad Kissingen, Germany) for genetic studies. Pedigrees for the core family were analyzed for common ancestry. A review of submissions to the Coagulation Laboratory was performed to identify samples from additional MCCs tested for FXI activity over a two-year period (January 2019 to January 2021). An archived DNA sample of a domestic shorthair cat was used to sequence *F11*. Archived DNA samples for MCCs and other cat breeds in Europe were genotyped for *F11* gene variants (Labogen; sent for routine genetic testing other than FXI deficiency). Genotyping studies were approved by the governmental animal care and use committee in Bavaria, Germany (RUF-55.2.2-2532-1-86-5).

### 2.2. Coagulation Assays

Activated partial thromboplastin time (aPTT) and prothrombin time (PT) assays, and FVIII, FIX, FXI, and FXII activities were measured in citrated plasma using STA-Compact and ST4 coagulation analyzers (Diagnostica Stago, Parsippany, NJ, USA) as described [25,26]. Briefly, human congenital factor deficient plasmas (George King Bio-Medical, Overland Park, KS, USA) were used in modified aPTT assays to measure the intrinsic factor activities. A feline pooled plasma was prepared in-house from 15 healthy domestic shorthair cats. Dilutions of the pooled plasma were used to generate factor assay standard curves. Factor activities were reported as the percentage activity of the pooled standard that had an assigned activity of 100% for each factor tested.

### 2.3. DNA Sequencing

The 14 coding exons and adjacent intronic regions of the feline *F11* gene were sequenced bidirectionally on an ABI Genetic Analyzer 3130 (Applied Bioscience, Thermo Fisher Scientific, Waltham, MA, USA). Template DNA was extracted from blood (GenElute^TM^ Blood Genomic DNA Kit; Sigma Aldrich, Merck KGaA, Darmstadt, Germany) and buccal swabs (MagNA Pure 96 Instrument; Roche Diagnostics GmbH, Penzberg, Germany). The primer pairs based on *F11* coding sequences from a reference genome for Felis catus 9.0 (NCBI accession no. XM_003984601.5) are listed in Supplemental Table S1 [27]. DNA sequences from MCCs were compared with the feline reference genome sequence. Predicted amino acid sequences for wild-type and variant FXI were analyzed for homology to other species (Clustal Omega [28]). The first methionine of the signal peptide was designated as amino acid number one. Web-based tools were applied to predict the impact of non-synonymous variants on the protein (PROVEAN, SIFT) [29,30].

### 2.4. Genotyping

DNA sequencing with primer pair 13 detected an *F11* missense variant (XM 003984601.5:c.1546G>A) in MCCs with FXI deficiency. The change resulted in substitution of FXI valine 516 (V516) with methionine (M516). A polymorphism within one of the primer binding sites was identified in some MCCs that caused inconsistent sequencing of the wild-type allele. A second primer pair (#13a) was generated (Supplemental Table S1), which properly sequenced the alleles. A TaqMan^®^ SNP Genotyping Assay was performed for the missense *F11* variant (Thermo Fisher Scientific) using Rotor-Gene 6000 (Corbett/QIAGEN, Hilden, Germany). The initial TaqMan^®^ primer pair (#13b) did not discriminate clearly between heterozygous and wild-type cats (missing the mutant *F11* allele in heterozygotes), so a second primer pair (#13c) was developed that reliably discriminated between the genotypes (Supplemental Table S1).

### 2.5. Factor XI Immunoprecipitation from Plasma

Citrated plasma from FXI-deficient and non-deficient humans, a baboon, and a domestic cat were tested. Anti-FXI monoclonal IgG 14E11 was linked to Affigel-10 beads (BioRad, Richmond, CA, USA) at 3 mg/mL [31]. One ml of plasma diluted 1:1 with Tris-buffered saline (TBS; 50 mM Tris-HCl pH 7.4, 100 mM NaCl) was incubated with 50 µL 14E11-beads at room temperature (RT) for two hours. Beads were washed with TBS and eluted with 50 µL sodium dodecyl sulfate (SDS)-non-reducing sample buffer. Eluates were size fractionated by SDS-polyacrylamide gel electrophoresis (PAGE), transferred to a nitrocellulose membrane, and incubated with biotinylated IgG 14E11. Detection was with streptavidin-horseradish peroxidase (Thermo Scientific) and chemiluminescence [32]. Western blots of citrated plasma samples (1 µL) and recombinant proteins were performed in a similar manner.

### 2.6. Recombinant FXI

cDNAs encoding wild-type feline and human FXI (fFXI-V516 and hFXI-V516, respectively) were synthesized based on reference genome sequences. Sequence encoding a nine amino acid hemagglutinin (HA) tag (YPYDVPDYA) was added to the C-termini. The cDNAs were introduced into mammalian expression vector pJVCMV [33]. The nucleotide triplet GTG encoding V516 in the fFXI cDNA was changed to ATG coding for methionine (fFXI-M516). 293 human embryonic kidney (HEK) fibroblasts (American Type Culture Collection [ATCC CRL 1573], Rockville, MD, USA) were transfected with 40 μg of pJVCMV-FXI constructs and 2 μg of pRSVneo, encoding a neomycin resistance marker using an Electrocell Manipulator 600 (BTX, San Diego, CA, USA). G418-resistant clones expressing G418-resistant FXI were expanded in Dulbecco’s modified Eagle medium with 5% fetal bovine serum and 500 μg/mL G418, then transferred to a serum-free medium. Recombinant FXI was purified by affinity chromatography using an anti-HA antibody (Invitrogen/Thermo Scientific, Waltham, MA, USA).

### 2.7. Factor XI Activity in aPTT Assays

Thirty microliters of recombinant FXI (0.3–30 nM) in TBS with 0.1% bovine serum albumin (TBSA) was mixed with 30 µL human FXI-deficient plasma and 30 µL PTT-A reagent (Diagnostica Stago, Asnières-sur-Seine, France). After incubation at 37 °C for 5 min, 30 µL of 25 mM CaCl_2_ was added, and time to clot formation was determined with an ST4 coagulation analyzer (Diagnostica Stago, Asnières-sur-Seine, France). Clotting times were plotted against FXI concentration on log–log plots, and activity of fFXI-M516 as a percentage of fFXI-V516 activity was calculated.

### 2.8. Factor XI Activation

All reactions were carried out in 50 mM 4(-2-hydroxyethyl)-1-piperazine-ethanesulfonic acid (HEPES, Sigma-Aldrich, St. Louis, MO, USA) pH 7.4, 125 mM NaCl, 1 mg/mL polyethylene glycol (PEG) 8000 was incubated at RT. *Autoactivation.* FXI (60 nM) was incubated with 10 μg/mL dextran sulfate (MW 6.5 to 10 kDa) and 500 μM L-pyroglutamyl-L-prolyl-L-arginine-p-nitroaniline (S-2366; Diapharma, West Chester, OH, USA). Changes in OD405 nm were continuously monitored on a spectrophotometer. 

*Activation by FXIIa or thrombin.* FXI (200 nM) was incubated with 20 nM human FXIIa (Enzyme Research, South Bend, IN, USA), or a combination of human thrombin (6 nM) and polyphosphate (4 μM of polymers of 200 to 1300 phosphate units) in microfuge tubes coated with PEG 20000. At various times, aliquots were removed into reducing SDS-sample buffer, size fractionated by SDS-PAGE, and stained with Coomassie blue (GelCode Blue, Pierce, Rockford, IL, USA).

### 2.9. Factor IX Activation by FXIa

FXI was incubated with FXIIa in TBS at 37 °C for 24 h to generate FXIa. Conversion of the 80 kDa FXI zymogen to the 45 kDa heavy chain and 35 kDa catalytic domain of FXIa was confirmed by SDS-PAGE (Supplemental Figure S1). Human FIX (200 nM) in 50 mM HEPES pH 7.4, 125 mM NaCl, 5 mM CaCl_2_, and 1 mg/mL PEG 8000 was incubated at RT with FXIa (2 nM) in PEG 20000-coated microfuge tubes. Aliquots were removed at various times and mixed with non-reducing SDS-sample buffer. Samples were size fractionated with non-reducing 10% SDS-PAGE and stained with Coomassie blue. Gels were analyzed by band densitometry using an Azure Biosystems C600 imager (Dublin, CA, USA).

### 2.10. Chromogenic Assay for FXIa Activity

FXIa (6 nM) was incubated with 50 to 2000 µM S-2366 in 20 mM HEPES pH 7.4, 0.1 M NaCl, and 0.1% PEG 8000 at RT. Generation of free p-nitroaniline (*p*NA) was measured by following changes in absorbance at 405 nm on a microtiter plate reader. *p*NA generation was calculated from OD changes at 405 nm using an extinction coefficient of 9920 OD units (mol/cm^3^). Inverses of *p*NA generation were plotted against inverses of S-2366 concentration. The X-intercept was 1/*K_m_*, the Y-intercept was 1/*V_max_*, and the *k_cat_* was derived from *V_max_*.

### 2.11. Statistical Analysis

Statistical analysis was performed using MS Office Excel (Microsoft Corp., Redmond, WA, USA) and the SPSS Statistics (version 26; IBM Corp., Armonk, NY, USA) software programs. All continuous data were assessed for normal distribution. Differences in coagulation factor activities and aPTTs were evaluated using a Kruskal–Wallis test [34]. The level of significance was set at *p* < 0.05.

## 3. Results

### 3.1. F11 Sequence Variants in Maine Coon Cats

**Figure 1:**
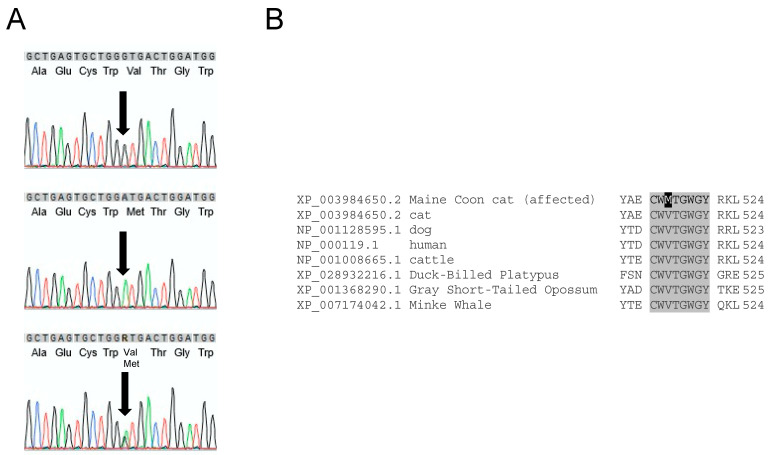
The FXI V516M substitution. (**A**) DNA and predicted amino acid sequence for a portion of exon 13 of the *F11* mRNA surrounding the single base substitution (XM_003984601.5:c.1546G>A) in Maine Coon cats. The top sequence is from a homozygote for wild-type fFXI-V516 (XP_003984650.2), the middle sequence from a homozygote for the missense variant fFXI-M516, and the bottom sequence from a heterozygote for fFXI-V516 and fFXI-M516. The top lines show the DNA codons in grey boxes, with the predicted amino acid sequences below. (**B**) Predicted FXI amino acid sequence alignments (Clustal Omega [28]) of the part of the FXI catalytic domain containing the V516M substitution. Shown are sequences from a MCC homozygous for the missense variant FXI-M516 (top) and several other mammals. Conserved amino acids are shaded in grey, and the missense variant in the FXI-deficient MCC is shaded black. Numbers on the right mark the last amino acid per row. NCBI database accession numbers are: XP_003984650.2 (feline), NP_001128595.1 (canine), NP_000119.1 (human), NP_001008665.1 (cattle), XP_028932216.1 (duck-billed platypus [*O. anatinus*]), XP_001368290.1 (gray short-tailed opossum [*M. domestica*]), and XP_007174042.1 (minke whale [*B. a. scammoni*]) [27]. All amino acid sequences are based on the initiator methionine in the signal peptide as the first amino acid. Note that the V516M is equivalent to V498M in the human Legacy FXI sequence commonly used for reporting human FXI mutations (EAHAD) in which the first amino acid of the mature plasma protein is designated as residue one [36].

The feline *F11* gene (XM_003984601.5) resides on chromosome B1 and contains fifteen exons. The predicted amino acid sequence (XP_003984650.2) is 85% identical to human FXI (NP_000119.1) (Supplemental Figure S2). We sequenced the 14 coding exons (exons 2–15) and adjacent intronic regions of *F11* from a domestic shorthair cat, 36 MCCs from the core family, and three archived DNA samples from non-related MCCs. All 13 MCCs in the core family with plasma FXI activity <30% of normal (and one with 42% FXI activity) were homozygous for a non-synonymous variant in exon 13 (XM003984601.5:c.1546G>A) (Figure 1A). Fourteen other MCCs were heterozygous for the variant. The substitution changed the first nucleotide of the triplet coding for valine 516 (V516), resulting in a methionine (M516) replacement (Supplemental Figure S2). V516 in human and feline FXI corresponds to V138 in the chymotrypsin numbering system used to compare trypsin-like serine proteases [35]. It is highly conserved in mammals including a monotreme, the duck-billed platypus (*Ornithorhynchus anatinus)* and resides in a conserved region of the catalytic domain (Figure 1B). Utilizing genetic variant impact tools, this missense variant was considered to be ‘neutral’ by PROVEAN but ‘not tolerated’ by SIFT [29,30].

In addition to V516M, nine synonymous variants and one non-synonymous variant were identified in *F11* exons of domestic shorthair cats and MCCs (Supplemental Table S2). A non-synonymous valine 8 to isoleucine variant was absent in affected MCCs and was not predicted to be deleterious to structure by PROVEAN or SIFT. Synonymous variants were dispersed over eight exons, and included a previously reported variant (Ensembl transcript: ENSFCAT00000001549.6 F11-201:c.282G>A; Supplemental Table S2) that was present in three MCCs. No synonymous variant segregated with V516M or low FXI activity.

### 3.2. Genotyping for FXI-V516M

**Figure 2:**
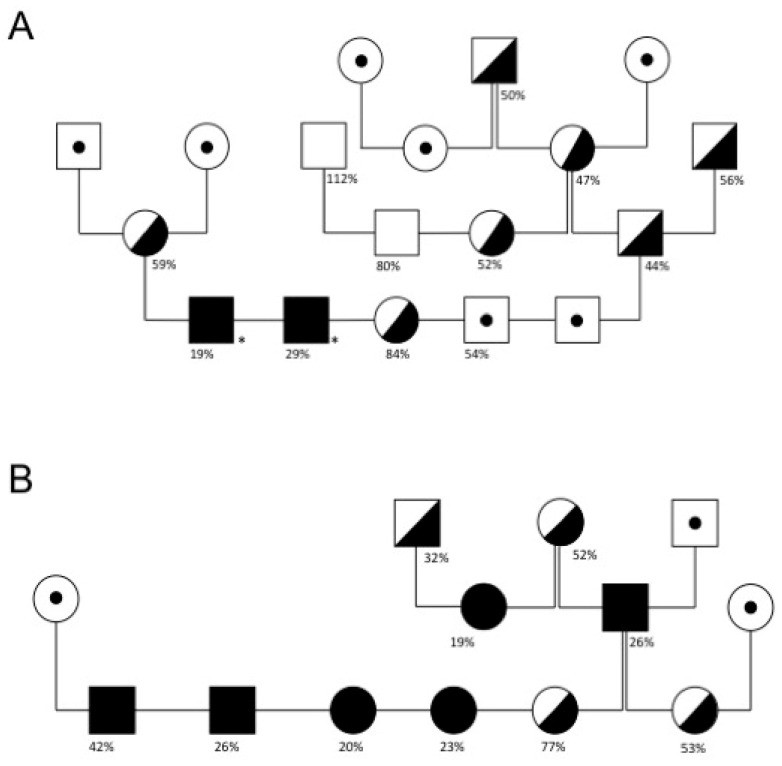
Maine Coon cat core family pedigrees. Pedigrees of the MCC core family from (**A**) the cattery of the index case and (**B**) a related cattery. FXI activity and excessive bleeding phenotype are indicated. Squares and circles represent males and females, respectively. Filled symbols indicate cats homozygous for the FXI-V516M with low FXI activity and/or prolonged aPTT. Half-filled symbols indicate cats that are carriers of the FXI-V516M variant. Percentage numbers below symbols indicate FXI activities. The black asterisk indicates animals with known clinical signs of bleeding. Symbols with a dot represent untested cats. Not all animals of the core family are shown in this pedigree.

A TaqMan^®^ SNP Genotyping Assay was developed to identify the FXI-V516M variant and confirm results of exon sequencing. Within the core family there were 13 homozygotes for FXI-V516M and 14 heterozygotes. While animals in the core family appeared to be closely related based upon pedigree analysis, and FXI-V516M segregated within the pedigree (Figure 2), a single common ancestor could not be identified for five generations spanning 17 years (DNA was not available for ancestral cats).

Archived DNA for 263 MCCs from Germany and some other European countries were genotyped for FXI-V516M. While their relatedness to the cats in the USA was not known (intercontinental breeding commonly occurs), 100 MCCs were found to be heterozygous (carriers), and 11 were homozygotes. Screening of 100 cats from 19 other breeds including 10 Norwegian Forest and 10 Siberian cats did not identify any cat with the V516M variant (Table 1).

### 3.3. Clinical Signs

Excessive bleeding was reported for eight MCCs homozygous for FXI-V516M and/or who had prolonged aPTTs with low plasma FXI activity (three from the core family, one from Italy and four identified by a retrospective analysis at Cornell University). Other related cats did not have histories of excessive bleeding. Episodic recurrent bleeding was reported at different ages (from juvenile up to 8 years of age at first presentation). Signs of abnormal hemostasis included easy bruising, gingival bleeding, bleeding with loss of deciduous teeth, subcutaneous and aural hematomas, and late post-operative bleeding. One animal experienced massive bleeding after being spayed. Another developed hematomas with recurrent bleeding despite transfusion support. Two MCCs (Figure 2A, marked with asterisks) had easy bruising. While we did not have clinical information for all animals, abnormal bleeding with trauma or surgery was reported only in MCCs homozygous for the V516M substitution.

### 3.4. Coagulation Test Results: aPTT and FXI Activity

**Figure 3:**
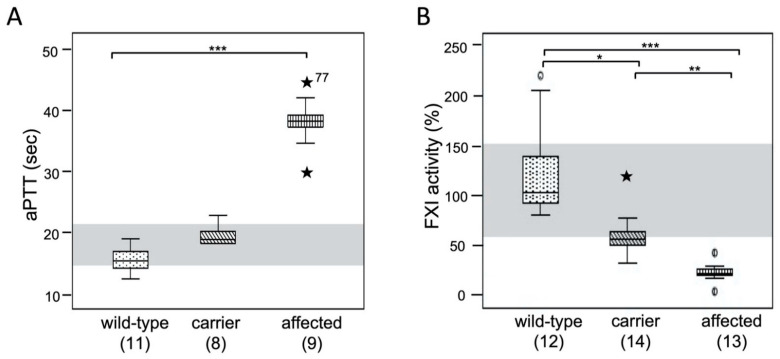
Coagulation test results. (**A**) Activated thromboplastin time and (**B**) plasma FXI activity, for MCCs of the core family based on genotyping results. The median, 95% confidence interval and range with outliers (★) are shown. The light grey horizontal bar indicates the aPTT (15.0–21.0 sec.) and FXI activity reference intervals (60–150%). *, *p* < 0.05; **, *p* < 0.01; ***, *p* < 0.001.

Coagulation screening tests including FXI activity assays were available for 39 core family MCCs. All MCCs homozygous for FXI-V516M substitution had prolonged aPTTs and normal PTs (Figure 3A and Supplemental Tables S3 and S4). Their aPTTs ranged from 30 to 42 sec (upper limit of normal 21 sec), except for one aPTT of 77 sec. The aPTTs of MCCs heterozygous for FXI-V516M were slightly prolonged when compared with homozygous wild-type MCCs (Figure 3A and Supplemental Tables S3 and S4 and).

Specific coagulation factor testing revealed reduced FXI activity (17–29% of feline pooled normal plasma) in plasmas of MCCs homozygous for FXI-V516M (Figure 3B, Supplemental Tables S3 and S4). Two MCCs homozygous for FXI-V516M with 3% (aPTT was not measured) and 42% FXI activities (prolonged aPTT [29 sec]) were at the extremes and were considered outliers ( Supplemental Tables S3 and S4). Cats heterozygous for FXI-V516M had aPTTs within or slightly above the reference interval, and normal to moderately reduced plasma FXI activities when compared with the pooled control plasma and wild-type MCCs. There was a strong correlation between genotype, aPTT, and FXI activity in MCCs (Figure 3A,B and Supplemental Tables S3 and S4).

A two-year retrospective case review at Cornell University identified an additional 72 MCCs (ages 0.2 to 8 years, equal gender distribution) from fourteen U.S. states with results for FXI activity testing. The median and range of FXI activity in these MCCs were 58% and 10–197%, respectively. Twelve cats had FXI activities <30%, suggestive of homozygosity for FXI deficiency, however, DNA was not available for confirmatory genotyping.

### 3.5. Factor XI in Feline Plasma

**Figure 4:**
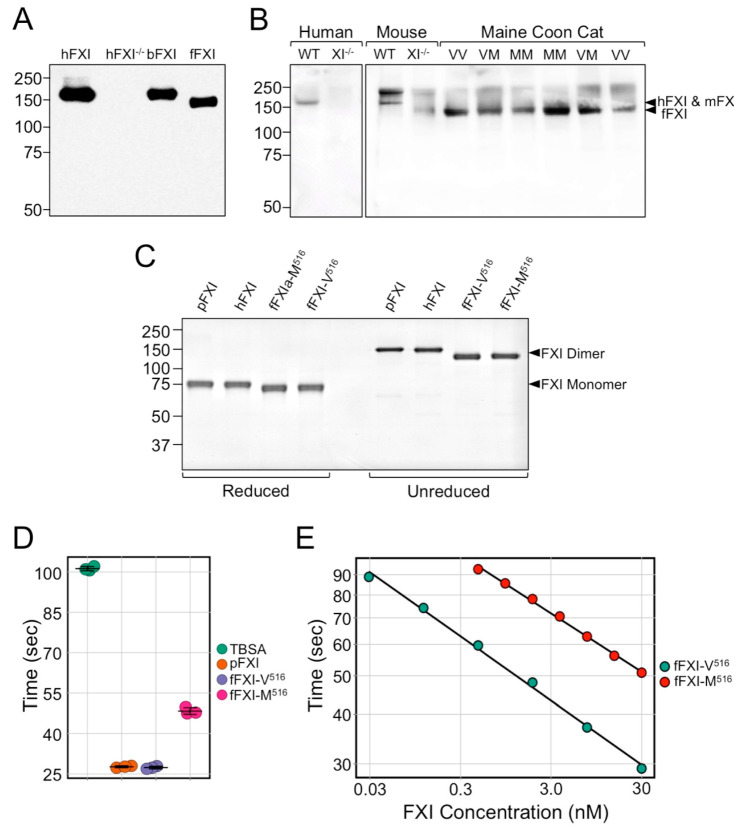
Plasma and Recombinant FXI. (**A**) Western blots of non-reducing 7.5% polyacrylamide gels of fXI immunoprecipitated from normal human plasma (hFXI), FXI-deficient human (hFXI^−/−^), baboon plasma (bFXI) and domestic cat plasma (fFXI) with 14E11 linked to agarose beads. The primary detection antibody was biotinylated-14E11. (**B**) Western blots of non-reducing 7.5% polyacrylamide gels of wild-type (WT) or FXI-deficient (XI^−/−^) human and mouse plasmas, and plasmas from MMCs homozygous for wild-type FXI (VV), homozygous for variant FXI (MM) or heterozygous for both forms of FXI (VM). The primary detection antibody was biotinylated-14E11. Positions of bands representing human (hFXI), mouse (mFXI) and feline (fFXI) are indicated on the right. (**C**) Coomassie blue stained 7.5% polyacrylamide gel run under reducing (left) or non-reducing (right) conditions of plasma-derived human FXI (pFXI); and recombinant wild-type human (hFXI), wild-type feline (fFXI-V516), and variant feline (fFXI-M516). Positions of the FXI monomer (reduced) and homodimer (unreduced) are indicated on the right. For panels A–C, positions of molecular mass standards in kDa are shown on the left. (**D**) aPTT clotting times for FXI-deficient human plasma supplemented with TBSA vehicle (TBSA), 30 nM plasma-derived human FXI (pFXI), 30 nM wild-type feline FXI (fFXI-V516), or 30 nM variant feline FXI (fFXI-M516). Reactions were run in triplicate. Horizontal bars indicate the means and one SD for each group. (**E**) Log-log plots of aPTT clotting times versus plasma FXI concentration for human FXI-deficient plasma supplemented with various concentrations of wild-type feline FXI (fFXI-V516) or variant feline FXI (fFXI-M516). The activity of the variant FXI as a percentage of wild-type FXI was determined, assuming the activity for 30 nM FXI-V516 was 100% of normal.

The antibody 14E11, which recognizes an epitope on the FXI A2 domain that is conserved in most placental mammals [1], immunoprecipitated a protein from the plasma of a domestic cat. The band ran slightly faster on SDS-PAGE than FXI from human and baboon (bFXI) plasmas (Figure 4A). As the FXI polypeptides from humans and cats both have 625 amino acids (Supplemental Figure S2), differences in post-translational modifications may explain the different migration. On Western blots of plasma, FXI in healthy MCCs also ran faster than human or mouse FXI (Figure 4B). Interestingly, FXI bands were present for MCCs homozygous for the M516 variant. This indicates the mutant protein is expressed and secreted by hepatocytes, forms a dimer, and is stable in plasma (i.e., a cross-reactive material positive [CRM+] FXI variant).

### 3.6. Factor XI Activity

We prepared recombinant fFXI-V516 and fFXI-M516 in HEK293 fibroblasts. The immunoblot (Figure 4C) confirmed that fFXI homodimers (unreduced) and monomers (reduced) migrated slightly faster than hFXI. To determine the relative activities of fFXI-V516 and fFXI-M516, we assumed that the FXI concentrations in feline and human plasmas were similar (~30 nM), based on the measured FXI activity of wildtype cat plasma (Figure 3B). The aPTT of human plasma completely lacking FXI was 101.2 ± 0.8 sec. Human FXI-deficient plasma supplemented with 30 nM fFXI-V516 or human plasma-derived FXI were 27.7 ± 0.4 and 27.4 ± 0.5 sec, respectively, while plasma supplemented with fFXI-M516 had a longer aPTT (48.4 ± 1.3 sec., Figure 4D). Using a modified aPTT assay in which human FXI-deficient plasma supplemented with 30 nM fFXI-V516 was assigned as FXI activity of 100% (Figure 4E), fFXI-M516 exhibited a markedly reduced activity (4% of control, Figure 4E).

### 3.7. Factor XI Activation

**Figure 5:**
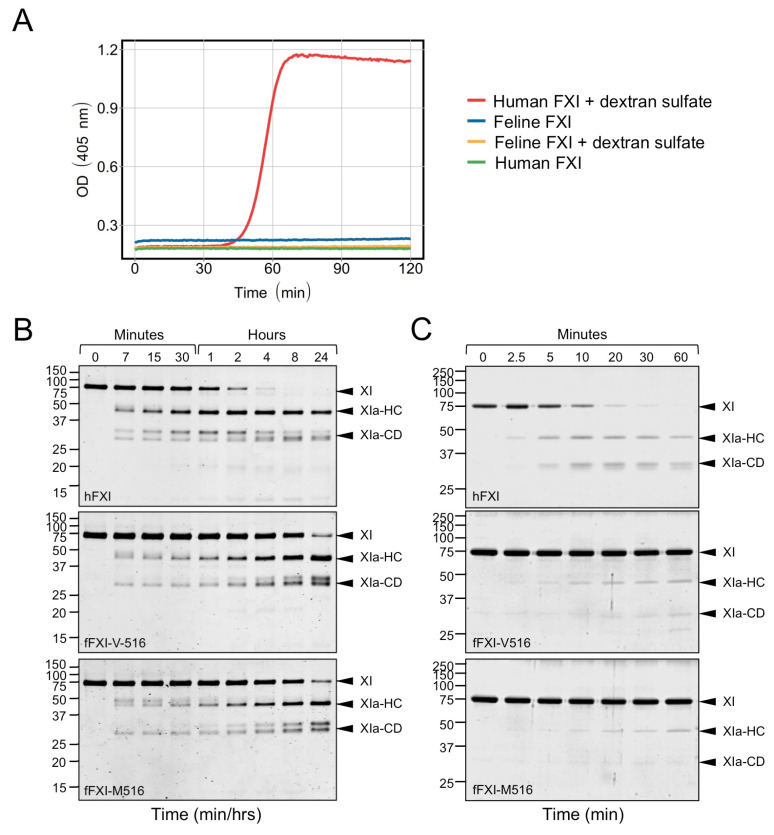
Factor XI activation. (**A**) Autoactivation of FXI. FXI (60 nM) was incubated with 10 μg/mL dextran sulfate (MW 6.5 to 10 kDa) and 500 μM S-2366. Changes in OD405 nm were continuously monitored on a spectrophotometer. (**B**) FXI activation by FXIIa. Time courses of 200 nM human wild-type FXI (hFXI), feline wild-type FXI (fFXI-V516), or feline variant FXI (fFXI-M516) incubated with 20 nM human FXIIa. (**C**) FXI activation by thrombin. Time courses of 200 nM human wild-type FXI (hFXI), feline wild-type FXI (fFXI-V516), or feline variant FXI (fFXI-M516) incubated with 6 nM human thrombin and 4 μM polyphosphate. For panels B and C, samples were removed into reducing SDS-sample buffer at the indicated times, then size fractionated on reducing 10% polyacrylamide gels and stained with Coomassie blue. Positions of standards for zymogen FXI (XI), and the heavy chain (HC) and catalytic domain (CD) of FXIa are indicated on the right.

Human FXI is converted to FXIa by autoactivation in the presence of a polyanion such as dextran sulfate. Interestingly, wild-type fFXI did not undergo autoactivation under similar conditions (Figure 5A). In aPTT assays, FXIIa must convert FXI to FXIa to initiate clotting. Human FXIIa converted fFXI-V516 and fFXI-M516 to FXIa at comparable rates, but more slowly than hFXI (Figure 5B). It is possible that fFXI is a poorer substrate for human FXIIa than hFXI. More importantly, the similar results for fFXI-V516 and fFXI-M516 indicated that a defect in activation is unlikely to be responsible for the poor performance of fFXI-M516 protein in the aPTT assay. The absence of a bleeding disorder associated with FXII deficiency implies that FXI may be activated by proteases other than FXIIa. Thrombin has been shown to activate FXI, and the reaction is enhanced by polyanions such as dextran sulfate [37]. With hFXI, conversion to FXIa in the presence of thrombin and dextran sulfate is due to a combination of thrombin-mediated proteolysis and autoactivation (Figure 5C, top image). Because fFXI does not undergo autoactivation, conversion of fFXI-V516 and fFXI-M516 to FXIa by thrombin and dextran sulfate was considerably slower than for hFXI (Figure 5C, middle and bottom images), with the two feline proteins activating similarly.

### 3.8. Factor XIa Activity in a Chromogenic Assay

**Figure 6:**
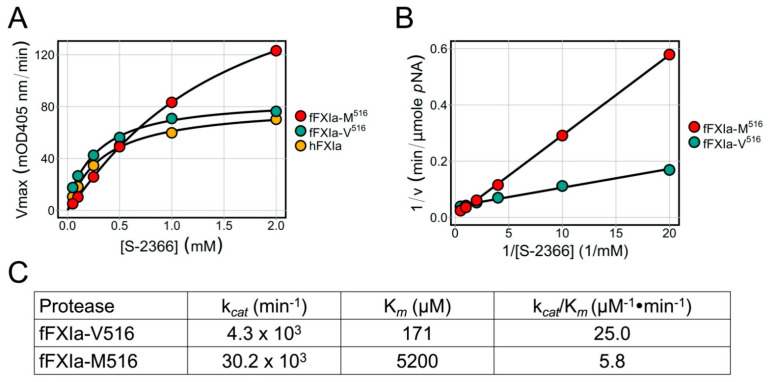
Chromogenic assays for FXIa activity (**A**) Six nanomolar human wild-type FXIa (hFXIa), feline wild-type FXIa (fFXIa-V516) or feline variant FXIa (fFXIa-M516) were incubated with varying concentrations of S-2366, and change in OD405 nm over time was followed on a microtiter spectrophotometer. (**B**) Inverse reciprocal plots for data in panel C. Values for OD405 nm/min were converted to *p*NA formed per unit time. (**C**) Kinetic parameters for S-2366 cleavage by FXIa derived from the reciprocal plots in panel B.

Using the tripeptide chromogenic substrate S-2366, hFXIa and fcFXIa-V516 displayed comparable activities across a range of S-2366 concentrations (Figure 6A). The fFXIa-M516 protein also displayed substantial activity, but with a distinctly different pattern than for hFXIa or fFXIa-V516 (Figure 6A). fFXIa-M516 actually turned S-2366 over faster than fFXIa-V516 at high substrate concentrations (higher *V_max_*), but with a substantially higher *K_m_* (Figure 6B,C) indicating poorer affinity for the substrate. The result was an ~4.3-fold reduction in catalytic efficacy (*k_cat_*/*K_m_*) for fFXI-M516 compared with fFXI-V516, supporting the hypothesis that the V516M substitution alters the conformation of the protease active site.

### 3.9. Factor IX Activation by FXIa

**Figure 7:**
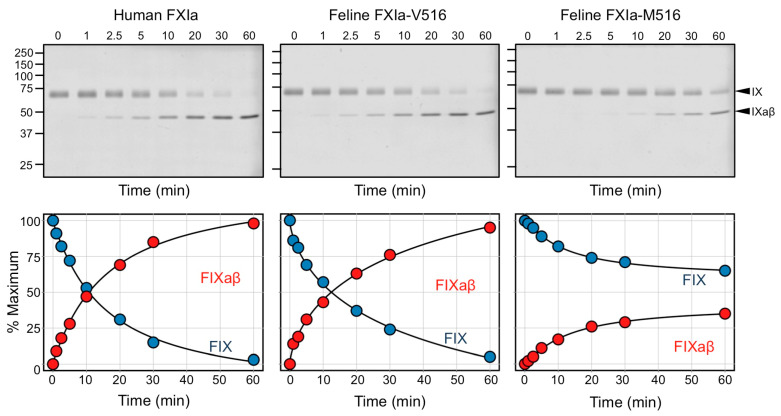
Factor IX Activation by FXIa. Time courses of 200 nM human FIX incubated with 2 nM human wild-type FXIa (hFXIa), feline wild-type FXIa (fFXIa-V516), and feline variant FXIa (fFXIa-M516). At the indicated times, samples were removed into non-reducing SDS-sample buffer. Samples were size fractionated on reducing 10% polyacrylamide gels and stained with Coomassie blue. Positions of standards for zymogen FIX (IX) and the protease FIXaβ (IXaβ) are indicated on the right. Below each gel are results of densitometric analyses, showing reduction in FIX and formation of FIXaβ as functions of time.

The primary function of FXIa during hemostasis is to convert zymogen FIX to the protease FIXaβ. This is accomplished by two sequential cleavages that release the FIX activation peptide [38]. In time course experiments, hFXIa and fFXIa-V516 converted human FIX to FIXaβ at comparable rates, while FIX activation by fFXIa-M516 was ~3.2-fold slower (Figure 7), consistent with a catalytic defect in fFXI-M516.

## 4. Discussion

The study of hereditary coagulopathies in domesticated animals can improve the diagnosis and treatment of affected individuals in veterinary medicine, and facilitate breeding strategies to prevent propagation of inherited diseases. Moreover, domestic animals are substantially larger than mice and their hereditary coagulopathies may serve better as translational models for understanding the pathogenesis and propagation of human bleeding disorders, and for assessing efficacy and safety of novel treatments. A variety of hereditary bleeding disorders, including deficiencies of factor VIII and factor IX, have been described in domesticated animals. In particular, hemophilic dogs have been used extensively as intermediates between mouse models and human patients to study and introduce novel therapies [39].

Approximately 400 *F11* variants associated with FXI deficiency in humans are listed in the new Factor XI Gene (*F11*) Variant Database European Association for Haemophilia and Allied Disorders (EAHAD, https://f11-db.eahad.org, accessed 26 March 2022) [36,40]. More than half are missense variants, and they are distributed over the entire *F11* gene. While the pathogenic effects of many of these variants have not been studied in detail, most are associated with a lack of variant protein in the circulation (CRM-deficiency). Few circulating FXI variants (CRM+ deficiency) have been described. We report here on an *F11* missense mutation in MCCs associated with a CRM+ FXI variant, reduced FXI activity in plasma, and a mild–moderate bleeding tendency. A single base change (XM 003984601.5:c.1546G>A) in the feline *F11* gene results in valine-516 in the trypsin-like catalytic (serine protease) domain of FXI being replaced with methionine (V516M). Residue 516 corresponds to amino acid 498 in numbering systems that designate the first amino acid of mature human plasma FXI as residue 1. The same FXI-V516M missense variant was described in a Korean woman with a bleeding disorder [41].

While this is the first report of a *F11* variant in FXI-deficient cats, *F11* insertions associated with bleeding disorders due to FXI deficiency have been reported in cattle and dogs. In Holstein–Friesian and Indian Sahiwal cattle, a 76 bp insertion introduces a termination codon, resulting in an mRNA encoding a protein lacking a functional catalytic domain (lacking exons 13–15), and premature mRNA decay [18,22]. In Japanese-Black cattle, a 15 bp insertion next to the cysteine-291 residue that stabilizes the structure of the fourth apple domain is predicted to disturb formation of the FXI homodimer [21,42,43]. Factor XI deficiency in Kerry Blue Terrier dogs is caused by a short interspersed nuclear element (SINE) insertion in exon 15 [23,24].

The V516M substitution replaces a highly conserved hydrophobic residue with another hydrophobic residue, albeit with a bulkier side chain. The normal migration and intense band for FXI in plasma on Western blots are consistent with a normal plasma FXI protein concentration in MCCs homozygous for the substitution, and indicate that FXI tolerates the V516M replacement without gross structural perturbation. However, our studies indicate that an alteration in the conformation of the protease active site reduces the rate of FIX activation. Activation of human FIX by FXIa or by the factor VIIa/tissue factor complex involves sequential cleavage of the FIX R145-A146 and R180-V181 peptide bonds. Activation goes through an intermediate, FIXα, in which only the R145-A146 bond is cleaved. FIXα accumulates during activation by factor VIIa/tissue factor because the second cleavage after R180 is rate limiting. In contrast, no intermediate accumulates during FIX activation by FXIa, because the initial cleavage at R145-A146 is rate limiting. The FXI activation mechanism depends on a FIX-binding exosite in the FXIa A3 domain, and its loss reduces the cleavage rates of both FIX bonds, and results in accumulation of FIXα. During FIX activation by fFXIa-M516, the rate of FIXaβ generation is reduced, but no FIXα accumulates, indicating that the relative rates of the two cleavages are maintained, and that the A3-domain dependent part of the activation mechanism is intact [2,44].

The bleeding tendency in MCCs from the core family is consistent with an autosomal recessive disorder (i.e., no clinical bleeding in heterozygotes). The size and amount of the FXI dimers found in heterozygous cats appeared similar to those in wild-type and affected cats. The observed plasma FXI activities were approximately half normal, detected as a mild prolongation of the aPTT but with no evidence for an increased bleeding tendency. However, as V516M is a CRM+ variant, and as FXI is a homodimer, heterozygotes for V516M likely have three forms of plasma FXI proteins: wild-type homodimers, mutant homodimers, and heterodimers with one wild-type and one mutant polypeptide. The effects of this on hemostasis may, therefore, not be reflected in the aPTT.

In humans, FXI deficiency is associated with a trauma- and surgery-related bleeding diathesis. However, the bleeding tendency is highly variable, and correlates poorly with plasma FXI activity. Indeed, many humans with severe FXI deficiency do not have a history of abnormal hemostasis and are identified serendipitously when an aPTT is performed. Others may experience abnormal hemostasis that occurs most often after trauma involving the mouth, nasopharynx, and urinary tract. Tissues in these areas are thought to have high fibrinolytic activity, leading to the hypothesis that FXI contributes to hemostasis by preventing premature fibrinolytic breakdown of clots [45]. Similarly, the bleeding tendency in FXI-deficient MCCs varies, with some asymptomatic animals (even after hemostatic challenge such as surgery) and others exhibiting mild–moderate bleeding with trauma or surgery. A similar bleeding diathesis has been reported in FXI-deficient Kerry Blue terrier dogs. FXI-deficient cattle have a mild bleeding tendency, with blood-tinged milk and prolonged bleeding from injection sites reported. Of greater importance for livestock production, FXI deficiency in cattle has been associated with reduced calving and increased susceptibility to infection [18]. Interestingly, total FXI deficiency has not been associated with hemostatic abnormalities in *F11* knockout mice [46]. It is conceivable that the contribution of FXI to hemostasis varies significantly between species [47].

In domesticated animals, mutant allele frequencies may reach high levels due to inbreeding practices for propagating desirable traits, limited regional breeding populations, and use of a limited number of sires that carry one or two copies of the mutant/variant allele. The frequency of the insertion, causing FXI deficiency in Holstein–Friesian cattle in Canada, was estimated to be between 8 and17% in 1980 [48]. Factor XI deficiency was first reported in Kerry Blue terriers in 1995, but while widely present there are no data on frequency. While there are no prior reports of common genetic variants causing FXI deficiency in feline breeds, our survey shows an unexpectedly high mutant allele frequency in MCCs in Europe. The large affected MCC core family and unrelated FXI-deficient MCCs identified in the retrospective review suggest that the FXI-V516M variant may be widespread among MCCs in the USA. A common ancestor was not identified for European and United States MCCs but pedigree analysis suggests that the variant likely originated within the last century. The frequent exchange of desirable breeding cats between countries explains the presence of the pathogenic variant on at least two continents. Genotyping in the USA was biased and focused on the core family, thus the frequency of the trait in the United States will require additional study, but it may well be similar to that in Europe (23.2%). The FXI-V516M variant was not found in any other feline breeds in the European samples genotyped, including Norwegian Forest and Siberian cats that are thought to be closely related to the MCC breed. However, our genotyping survey of other cat breeds was limited. There is one prior report of a bleeding domestic shorthair cat with FXI deficiency, however molecular genetic analysis was not performed [49].

Factor XI deficiency in MCCs has important clinical ramifications. FXI deficiency should be included in the differential diagnosis for any MCC with unexplained and abnormal bleeding. However, hemophilia A and B must be considered if bleeding is severe, particularly in a male cat, as spontaneous new mutations in *F8* and *F9* do occur and are also characterized by prolongation of the aPTT. Finally, FXII deficiency should also be considered in cats with an isolated aPTT prolongation, particularly if there is no history of excessive bleeding. FXII deficiency is common in domestic shorthair cats and has been reported in numerous other breeds including MCCs [25]. Recently, a fibrinolytic defect was reported in several bleeding MCCs from the United Kingdom, but the molecular defect remains unknown [50].

Diagnosis of FXI deficiency in MCC with clinical signs of bleeding has therapeutic relevance. Treatment of coagulation factor deficiencies in cats is restricted to fresh whole blood or fresh frozen plasma transfusion due to the small unit size that can be collected from a cat (typically 50 mL). While FXI-deficient humans may be treated with plasma or FXI-concentrate, antifibrinolytic drugs such as tranexamic acid and ε-aminocaproic acid have become the mainstay of therapy [51]. FXI-containing blood products were used successfully in FXI-deficient dogs [23]. Furthermore, use of antifibrinolytic agents appears to be safe in dogs [52,53,54], and could be tested in FXI-deficient dogs. However, cats have relatively restricted drug metabolisms and are prone to adverse side-effects with many drugs (e.g., acetylsalicylic acid). The effects and safety of antifibrinolytics has not been studied in cats.

Surgical bleeding in FXI-deficient animals can be avoided with pre-operative transfusion or managed through surgical technique and close post-operative monitoring. It seems advisable to screen MCCs with aPTT tests or *F11* genotyping prior to surgery. Indeed, screening for FXI-V516M could be performed along with screening for other known deleterious traits in the breed as part of wellness, pre-sale, or pre-breeding exams. It is often desirable to identify hereditary diseases in domestic animals to avoid breeding affected individuals. However, other important considerations are the available gene pool (which may be very narrow), maintenance of desirable traits, and the severity of a particular disease and its manageability. In this regard, the generally mild and manageable clinical phenotype of FXI deficiency may be less of a consideration than other disease traits in MCCs such as hypertrophic cardiomyopathy, erythroid pyruvate kinase deficiency, and spinal muscular dystrophy.

## 5. Conclusions

In conclusion, we present the first clinical phenotype to molecular genetic characterization of FXI deficiency in cats. fFXI-M516 is a CRM+ variant with impaired catalytic activity that reduces the capacity of FXIa to activate FIX. It appears to be common in the MCC breed and causes a mild–moderate bleeding tendency. Genotyping for *F11* variant along with other breed specific pathogenic variants is recommended for MCCs.

## Figures and Tables

**Table 1:** Comparison of *F11* mutant allele frequencies in Maine Coon cats from the USA and Europe, and other cat breeds in Europe assessed by Taqman^®^ SNP Genotyping Assay.

Cats	Total Number #	*F11* Genotype (XP_003984650.1:p.V516M)			Mutant (M516) Allele Frequency
		VV	VM	MM	
MCCs from USA	39	12	14	13	0.51
MCCs Europe	263	152	100	11	0.23
Other Feline Breeds *	100	100	-	-	0.00

* 17 breeds; VV, wild-type; VM, heterozygous; MM, homozygous mutant.

## Data Availability

Not applicable.

## Supplementary Materials

The following supporting information can be downloaded at: https://www.mdpi.com/article/10.3390/genes13050792/s1, Table S1: Primer sequences; Table S2: Variants in sequenced Maine Coon cats; Table S3: Hemostatic test results; Table S4: Comparison of coagulation screening tests and specific factor activities; Figure S1: Incubation of FXI with FXIIa; Figure S2: Amino acid sequences of human and feline FXI.
