# Supplementary material for: A Common Missense Variant Causing Factor XI Deficiency and Increased Bleeding Tendency in Maine Coon Cats
Source: Genes (Basel). 2022 Apr 28;13(5):792. doi: 10.3390/genes13050792 (PMC9140718; doi:10.3390/genes13050792)
Supplement: Supplementary file 1 [file genes-13-00792-s001.zip › genes-1680707-supplementary.pdf]

Original Article

A common missense variant causing factor XI deficiency and increased bleeding tendency in Maine Coon cats

SUPPLEMENTAL TABLES AND FIGURES

Supplemental Table S1: Primer sequences. Primer sequences used for DNA amplification and sequencing of the coding regions of feline *F11* exons and adjacent regions, and primer sequences for the TaqMan® SNP Genotyping Assay of the feline *F11* gene. Primer pair 13a and 13c were used to reliably discriminate the three genotypes (Primer pair 13 and 13b had a primer binding site mutation).

Exon	Binding Site	Primer Sequence	Amplicon Size (bp)
2	5' UTR	F: 5'-CTCCTCTCCCTCCCTGTTTC-3'	250
	Intron 2	R: 5'-GGTGTGGGCCTGTAGATCAG-3'	
3	Intron 2	F: 5'-ACCTACTTCGCTTGCCTTTG-3'	302
	Intron 3	R: 5'-AATCATCCCAGAAAGCTACTCC-3'	
4	Intron 3	F: 5'-TTGTTTTGGCATCAGATAGAGC-3'	303
	Intron 4	R: 5'-CGCAGGGCTTTGTTTCATAG-3'	
5	Intron 4	F: 5'-AGGAAGGGCAGTTGCTAACG-3'	338
	Intron 5	R: 5'-AAATTCTGGCATCAAGTCGAG-3'	
6	Intron 5	F: 5'-CGTATCGTCTCATGTTGTTTCC-3'	274
	Intron 6	R: 5'-GCACGTGGGTGTCCTAAGTC-3'	
7	Intron 6	F: 5'-TTGGGGAGTTGATTTCTTAAAC-3'	301
	Intron 7	R: 5'-GCAGACATTTCGATTAAACGTG-3'	
8	Intron 7	F: 5'-TCCATTTATATTCCCTCCTCTCC-3'	258
	Intron 8	R: 5'-CTAGGCAGGAAAGCACACATC-3'	
9	Intron 8	F: 5'-CCTGATCAAGGTCAACAAACC-3'	328
	Intron 9	R: 5'-GCAGGTGGGAAACAGAAGAG-3'	
10	Intron 9	F: 5'-GATACTCCCATCCCGTCTCC-3'	267
	Intron 10	R: 5'-CCCCTTTGCGGTTACTATTG-3'	
11	Intron 10	F: 5'-ATCGAGGGCAGGTTTTCTTC-3'	343
	Intron 11	R: 5'-TTACTAAAATGCTAATGACAGATTCAG-3'	
12	Intron 11	F: 5'-AATGTTTGGGAATCATTTGG-3'	328
	Intron 12	R: 5'-GGAGACTGTGGGGCTCAG-3'	
13	Intron 12	F: 5'-CCTGGGAGAGAAGATGTATTTTG-3'	253
	Intron 13	R: 5'-CACATTTTAGCTGTGGGACTTG-3'	
13a	Intron 12	F: 5'-TGAATGACGATGAGGCACAG-3'	253
	Intron 13	R: 5'-CAAGTGAGGAGGATCCCATG-3'	
14	Intron 13	F: 5'-TTTTTGTATCGGTACGCTTTTG-3'	329
	Intron 14	R: 5'-ACAAATTGACCGTGGTTCC-3'	
15	Intron 14	F: 5'-AAATCACTCTGAGACCCATGC-3'	350
	3'- UTR	R: 5'-GCTCGGTGTCTGTCTCCTTC-3'	
		**Original and Optimized TaqMan® SNP Genotyping Assay**	
13b	Intron 12	F: 5'-CAAAGGAGATCGAAGCGTAATATATGC-3'	87
	Intron 13	R: 5'-CAGAAGCATGGTTTCTTACCTCCTA-3'	
13c	Intron 12	F: 5'-CAAAGGAGATCGAAGCGTAATATATGC-3'	83
	Intron 13	R: 5'-GCATGGTTTCTTACCTCCTAATTTTCTG-3'	

bp, base pairs; F, forward primer; R, reverse Primer; UTR, untranslated region; VICTM, Dye label at 5' end (Thermo Fisher Scientific); FAMTM, Dye label at 5' end (Thermo Fisher Scientific). VICTM, 5'-AGTGCTGGGTGACTGG-3' Dye label at 5' end and FAMTM, 5'-AGTGCTGGATGACTGG-3' Dye label at 5' end (Thermo Fisher Scientific) used for both assays.
Standard PCR protocol was used: 1) Pre- Denaturation (95°C, 5 min); 2) Denaturation (95°C, 0.5 min); 3) Annealing (59.5°C, 0.5 min); 4) Elongation (72°C, 1 min); 5) Denaturation 95°C, 45 sec); 6) Annealing (59.5°C, 45 sec); 7) Elongation (72°C, 45 sec); 8) Final elongation (72°C, 10 min); and 9) Cooling (4°C, ~2 hours). Step 2-4 (9 cycles), and step 5-7 (30 cycles).

Supplemental Table S2: Variants in sequenced Maine Coon cats. Variants in sequenced Maine Coon cats compared to the published coding exons and sequence of the feline genome and the sequence of a domestic shorthair cat.

Exon	c. DNA position	p. position	SIFT analysis
2	23 G>T	V8I	tolerated
3	199 A>G	S66S	synonymous
4	282 G>A*	A94A*	synonymous
5	430 T>C	D143D	synonymous
6	574 A>G	K191K	synonymous
9	948 T>C<br>979 C>T<br>1000 A>G	A316A<br>R325R<br>S332S	synonymous<br>synonymous<br>synonymous
11	1297 T>C	C432C	synonymous
13	1547 G>A	V516M	not tolerated
14	1669 T>G	N556N	synonymous

* Previously reported, reference sequences used: feline amino acid reference sequenceV8I (XP_003984650.2), feline mRNA sequence (XM_003984601.5)

Supplemental Table S3: Hemostatic test results. Hemostatic test results of 13 FXI-deficient Maine Coon cats homozygous for the FXI-V516M substitution.

**Case #**	**PT (sec)**	**aPTT (sec)**	**FVIII (%)**	**FIX (%)**	**FXI (%)**	**FXII (%)**
3	ND	ND	>500	259	29	217
6	18.9	39.0	246	147	19	212
17	20.4	76.9	167	237	20	52
18	ND	ND	97	97	23	84
19	17.9	41.8	55	55	21	55
20	16.4	38.6	69	70	17	66
25	ND	ND	76	51	3	44
26	17.3	29.7	304	178	42	220
28	17.5	38.0	110	93	26	121
29	17.4	38.0	171	152	26	209
31	16.7	37.1	76	74	23	102
32	16.2	34.5	87	76	20	105
34	ND	ND	ND	ND	19	ND
**Median**	17.4	38.0	104	95	21	104
**Range**	(16.2-20.4)	(29.7-76.9)	(55-500)	(51-259)	(3-42)	(44-220)
**(n)**	(9)	(9)	(12)	(12)	(13)	(12)
**Reference Interval**	15.0-20.0	15.0-21.0	50-200	50-150	60-150	60-150

ND, not determined; (n), total number of animals tested.

Supplemental Table S4: Comparison of coagulation screening tests and specific factor activities in Maine Coon cats with different *F11* genotypes encoding residue 516.

Assay	Reference Interval	*F11* Genotypes		
		VV	VM	MM
**PT (sec)**	15.0-20.0	17.7, 18.9 (2)	18.0 (17.0-19.1) (5)	17.4 (16.2-20.4) (9)
**aPTT (sec)**	15.0-21.0	15.6 (12.7-19.1) (11)[a]	19.0 (18.3-22.9) (8)[ab]	38.0 (29.7-76.9) (9)[b]
**FVIII (%)**	50-200	125, 112 (2)	74 (64-291) (7)	104 (55-500) (12)
**FIX (%)**	50-150	142, 98 (2)	77 (71-195) (7)	95 (51-259) (12)
**FXI (%)**	60-150	103 (80-221) (12)[a]	56 (32-121) (14)[b]	21 (3-42) (13)[c]
**FXII (%)**	60-150	135, 85 (2)	100 (76-200) (11)	104 (44-220) (12)

VV, wild-type; VM, heterozygous; MM, homozygous mutant *$p < 0.05$; ** $p < 0.01$ compared to wild-type and $p < 0.05$ compared to heterozygotes. None of the values are normally distributed (median and range). Between wild-type and carrier as well as for carrier and affected, significant differences can be noted for the aPTT. For the FXI activity significant differences can be noted between all three groups.
Results with different letter superscripts (a, b, c) in the same line are significantly different from each other.

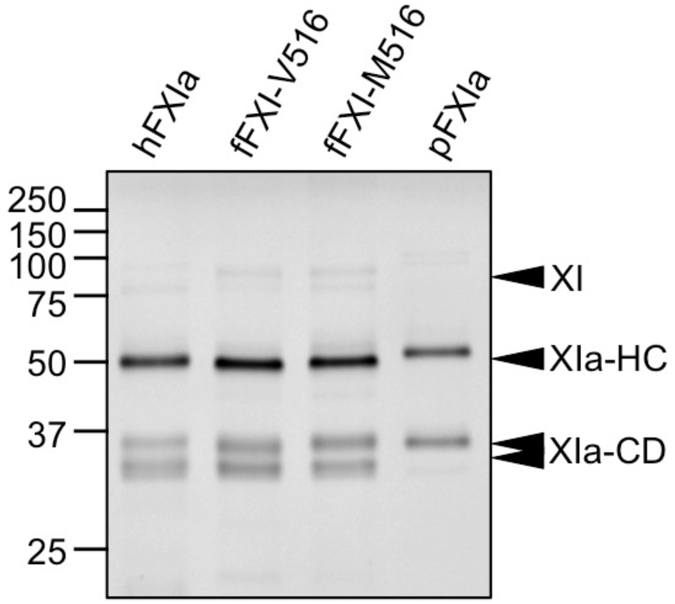

Supplemental Figure S1: Incubation of FXI with FXIIa. FXI was incubated with FXIIa (Enzyme Research, South Bend, IN) in TBS at 37°C for 24 hours to generate FXIa. Conversion of the 80 kDa FXI zymogen to the 45 kDa heavy chain and 35 kDa catalytic domain of FXIa was confirmed by SDS-PAGE. Shown is a Coomassie blue stained 10% polyacrylamide gel for two µg samples of recombinant human wild-type FXIa (hFXIa), wild-type cat FXIa (fFXIa-V516), feline variant FXIa (fFXIa-M516) and plasma-derived human FXIa (pFXIa). Positions of molecular mass standards in kDa are shown on the left, and markers for zymogen FXI (XI) and the heavy chain (HC) and catalytic domain (CD) of FXIa are indicated on the right. It is not clear why recombinant FXIa preparations have a doublet in the area where the CD runs, but this is a consistent finding.

```
         1                 *
Human    MIFLYQVVHFILFTSVSGECVTQLLKDTCFEGGDITTVFTPSAKYCQVVCTYHPRCLLFT
Cat      MILLYRVVHFILFASVSSECVTKLFTDTGFQGGDVTTIFSPSAKHCQLICTHHPRCLLFT

        61
Human    FTAESPSEDPTRWFTCVLKDSVTETLPRVNRTAAISGYSFKQCSHQISACNKDIYVDLDM
Cat      FMADSSSEDPTRWFTCILKDSVTETLPRVNMTGAISGYSFKQCPHQISACHRNLYVDLDM

       121
Human    KGINYNSSVAKSAQECQERCTDDVHCHFFTYATRQFPSLEHRNICLLKHTQTGTPTRITK
Cat      KGMNYNSLMTRDAQECQERCTNDVHCHFFTYATRQFPSTEHRNICLLKYTHMGTPTRIMK

       181
Human    LDKVVSGFSLKSCALSNLACIRDIFPNTVFADSNIDSVMAPDAFVCGRICTHHPGCLFFT
Cat      LTKVVSGFSLKSCALSKLACIRDIFPSTAFADSNIDSVMAPDVFVCRRICTHHPGCLFFT

       241
Human    FFSQEWPKESQRNLCLLKTSESGLPSTRIKKSKALSGFSLQSCRHSIPVFCHSSFYHDTD
Cat      FLSQEWPKESERNLCLLKTSESGLPSTRIKKDRALSGFSLQTCRHSVPVFCHSSFYHDTD

       301                                   339
Human    FLGEELDIVAAKSHEACQKLCTNAVRCQFFTYTPAQASCNEGKGKCYLKLSSNGSPTKIL
Cat      FLGEELDIVDVKGHEVCQKMCTDSIRCQFFTYSPSPESCNGGKGKCYLKLSSNGSPTKIL

       361                        387
Human    HGRGGISGYTLRLCKMDNECTTKIKPRIVGGTASVRGEWPWQVTLHTTSPTQRHLCGGSI
Cat      HGRGGISGYTLRLCKMDNVCTTKIKPRIVGGEASVHGEWPWQITLHITSPAQRHLCGGSI

       421       431                                              480
Human    IGNQWILTAAHCFYGVESPKILRVYSGILNQSEIKEDTSFFGVQEIIIHDQYKMAESGYD

Cat      IGNQWILTAAHCLIGLESPKILRVYSGILNQSEVKKDTAFFGVQEIIIHDQYEMAESGYD

       481                                 516
Human    IALLKLETTVNYTDSQRPICLPSKGDRNVIYTDCWVTGWGYRKLRDKIQNTLQKAKIPLV
Cat      IALLKLETAMNYTDAQRPICLPSKGDRSVIYAECWVTGWGYRKLGDKIQNTLQKANVPLV

       541                                575
Human    TNEECQKRYRGHKITHKMICAGYREGGKDACKGDSGGPLSCKHNEVWHLVGITSWGEGCA
Cat      TTEECQTRYRGHKITNKMLCAGYQEGGKDACKGDSGGPLSCKHNDVWHLVGITSWGEGCG
         *
       601                      625
Human    QRERPGVYTNVVEYVDWILEKTQAV
Cat      QRERPGVYTNVVEYVDWILEKTQAV
```

Supplemental Figure S2: Amino acid sequences of human and feline FXI. Shown are predicted amino acid sequences for human (Homo sapiens) and feline (Felis catus) FXI. The Initiator methionine is designated residue 1. The first amino acid of the mature FXI protein in plasma is glutamine 19 (E19 indicated by asterisk). Cysteine residues involved in disulfide bonds are highlighted in black. Cysteine 339 forms an interchain disulfide bond that connects the two polypeptides of the FXI homodimer. Valine 516 is substituted in affected Maine Coon cats (highlighted in green). The residues of the catalytic triad (H431, D480, and S575) are highlighted in red, and the activation cleavage site at arginine 387 is highlighted in blue.
